# Errata

**Published:** 2008-12

A typographical error on page A480 of the November Focus article [“Linking TB and the Environment: An Overlooked Mitigation Strategy,” *Environ Health Perspect* 116:A478–A485 (2008)] misidentified DOTS as a 66-month treatment regimen. DOTS is actually a 6-month regimen. *EHP* regrets the error.

Brauer et al. [*Environ Health Perspect* 116:680–686 (2008)] identified several minor errors in their recent publication. First, in the “Results” section of the abstract, the values given for low full-term birth weight (LBW) were incorrect; “Residence within 50 m of highways was associated with … a 22% (95% CI, 0.81–1.87) increase in LBW.” There were also inconsistencies in the description and application of missing data rules across all pollutants (Tables 3–7); the description of these rules in the text (bottom of p. 681) should read “For both approaches, a subject was considered missing if there was a gap of > 5 consecutive days in air monitoring data or if there were > 10 missing days within the term of the pregnancy.”

In Tables 2, 3, and 5, results for the small-for-gestational-age (SGA) outcome were presented for births restricted to gestational periods of ≥37 weeks, and not for the full cohort; in Tables 2, 3, 5, and 7, coding errors in the application of birth weight and gestational period cutoffs resulted in slight differences in the number of cases of SGA and preterm births; and in Tables 5 and 6, one road proximity measure was not labeled correctly: < 150 m from a major road/highway was actually 50–150 m from a major road/highway.

Correcting these errors results in small changes in the numbers of subjects included in specific analyses and slight differences in odds ratios (ORs) but does not alter our overall findings. The corrected tables are presented below.

The authors apologize for the errors.

**Table 2:** Prevalence of SGA, LBW and pre-term birth in the cohort (*n* = 70,249).

Outcome	No. (%)
SGA (< 10th percentile)	7,217 (10.3)
LBW (< 2,500 g at ≥37 weeks^a^)	897 (1.3)
Gestation < 37 weeks	3,748 (5.3)
Gestation < 35 weeks	1,247 (1.8)
Gestation < 30 weeks	241 (0.3)

a *n* = 66,501 births at full (≥37 weeks) term.

**Table 3:** Crude and adjusted ORs^a^ for SGA.

Exposure (entire pregnancy)	SGA cases (*n*)	Crude OR (95% CI)	Adjusted^b^ OR (95% CI)
NO–IDW (10 μg/m^3^)	7,215	1.02 (1.00–1.05)	1.05 (1.02–1.08)
NO–LUR (10 μg/m^3^)	7,023	1.03 (1.01–1.05)	1.03 (1.00–1.05)
NO_2_–IDW (10 μg/m^3^)	7,215	1.12 (1.08–1.16)	1.14 (1.09–1.18)
NO_2_–LUR (10 μg/m^3^)	7,023	1.04 (0.99–1.10)	0.98 (0.93–1.04)
CO–IDW (100 μg/m^3^)	5,779	1.04 (1.01–1.06)	1.06 (1.03–1.08)
PM_2.5_–IDW (1 μg/m^3^)	5,818	1.01 (0.97–1.05)	1.06 (1.00–1.12)
PM_2.5_–LUR (1 μg/m^3^)	7,023	1.03 (1.02–1.05)	1.01 (1.00–1.03)
BC–LUR (10^−5^/m)	7,023	1.03 (1.01–1.05)	1.01 (0.99–1.03)
PM_10_–IDW (1 μg/m^3^)	7,217	1.02 (1.00–1.04)	1.03 (1.00–1.07)
SO_2_–IDW (1 μg/m^3^)	7,138	1.00 (0.99–1.02)	1.01 (1.00–1.02)

BC, black carbon.

a ORs per standardized increases (indicated in parentheses), roughly corresponding to interquartile range.

b Adjusted for infant sex, First Nations status, parity, maternal age, maternal smoking during pregnancy, month–year of birth, income (quintile-census), maternal education (quartile-census).

**Table 4:** Crude and adjusted ORs^a^ for LBW at full (≥37 weeks) term.

Exposure (entire pregnancy)	LBW cases (*n*)	Crude OR (95% CI)	Adjusted^b^ OR (95% CI)
NO–IDW (10 μg/m^3^)	897	1.02 (0.96–1.09)	1.03 (0.96–1.11)
NO–LUR (10 μg/m^3^)	877	1.03 (0.97–1.09)	1.02 (0.95–1.08)
NO_2_–IDW (10 μg/m^3^)	897	1.11 (1.02–1.22)	1.10 (1.00–1.22)
NO_2_–LUR (10 μg/m^3^)	877	1.02 (0.89–1.18)	0.93 (0.80–1.08)
CO–IDW (100 μg/m^3^)	730	1.03 (0.97–1.09)	1.03 (0.96–1.11)
PM_2.5_–IDW (1 μg/m^3^)	724	0.96 (0.87–1.07)	1.00 (0.85–1.17)
PM_2.5_–LUR (1μg/m^3^)	877	1.05 (1.02–1.09)	1.03 (0.99–1.07)
BC–LUR (10^−5^/m)	877	1.03 (0.97–1.09)	1.01 (0.95–1.07)
PM_10_–IDW (1 μg/m^3^)	897	1.01 (0.95–1.08)	1.04 (0.95–1.13)
SO_2_–IDW (1 μg/m^3^)	891	0.99 (0.96–1.02)	0.98 (0.96–1.01)

BC, black carbon.

a ORs per standardized increases (indicated in parentheses), roughly corresponding to interquartile range.

b Adjusted for infant sex, First Nations status, parity, maternal age, maternal smoking during pregnancy, month–year of birth, income (quintile-census), maternal education (quartile-census).

**Table 5:** Crude and adjusted ORs for SGA and road proximity.

Measure	No. of SGA cases (% of total subjects)	Crude OR (95% CI)	Adjusted^a^ OR (95% CI)
< 150 m highway^b^ or < 50 major road^c^	1,374 (1.96)	1.04 (0.98–1.11)	1.02 (0.96–1.08)
< 50 m highway	176 (0.25)	1.25 (1.07–1.47)	1.21 (1.03–1.42)
50–150 m highway	404 (0.57)	0.95 (0.86–1.06)	0.92 (0.83–1.03)
< 50 m major road	841 (1.20)	1.06 (0.98–1.14)	1.04 (0.97–1.13)
50–150 m major road	1,592 (2.27)	1.05 (0.99–1.11)	1.04 (0.98–1.10)

a Adjusted for infant sex, First Nations status, parity, maternal age, maternal smoking during pregnancy, month–year of birth, income (quintile-census), maternal education (quartile-census).

b DMTI type 1 and 2 road (expressway: 114,000 vehicles/day; principal highway: 21,000 vehicles/day).

c DMTI type 3 and 4 road (secondary highway: 18,000 vehicles/day; major road: 15,000 vehicles/day).

**Table 6:** Crude and adjusted odds ratios for LBW at full term and road proximity.

Measure	No. of LBW cases (% of total subjects)	Crude OR (95% CI)	Adjusted^a^ OR (95% CI)
< 150 m highway^b^ or < 50 major road^c^	169 (0.25)	1.03 (0.87–1.22)	0.99 (0.84–1.18)
< 50 m highway	24 (0.04)	1.37 (0.91–2.07)	1.28 (0.85–1.93)
50–150 m highway	55 (0.08)	1.05 (0.80–1.39)	1.01 (0.77–1.33)
< 50 m major road	100 (0.15)	1.00 (0.81–1.24)	0.99 (0.80–1.22)
50–150 m major road	184 (0.28)	0.95 (0.81–1.12)	0.94 (0.80–1.10)

a Adjusted for infant sex, First Nations status, parity, maternal age, maternal smoking during pregnancy, month–year of birth, income (quintile-census), maternal education (quartile-census).

b DMTI type 1 and 2 road (expressway: 114,000 vehicles/day; principal highway: 21,000 vehicles/day).

c DMTI type 3 and 4 road (secondary highway: 18,000 vehicles/day; major road: 15,000 vehicles/day).

**Table 7:** Crude and adjusted ORs^a^ for preterm births < 30 weeks.

Exposure (entire pregnancy)	Cases (*n*)	Crude OR (95% CI)	Adjusted^b^ OR (95% CI)
NO–IDW (10 μg/m^3^)	241	1.15 (1.03–1.28)	1.18 (1.03–1.35)
NO–LUR (10 μg/m^3^)	235	1.09 (0.98–1.21)	1.06 (0.94–1.19)
NO_2_–IDW (10 μg/m^3^)	241	1.12 (0.94–1.33)	1.10 (0.91–1.34)
NO_2_–LUR (10 μg/m^3^)	235	1.15 (0.88–1.50)	1.09 (0.82–1.45)
CO–IDW (100 μg/m^3^)	192	1.06 (0.94–1.19)	1.04 (0.91–1.20)
PM_2.5_–IDW (1 μg/m^3^)	200	1.22 (1.01–1.48)	2.01 (1.38–2.92)
PM_2.5_–LUR (1μg/m^3^)	235	1.07 (0.99–1.14)	1.05 (0.97–1.13)
BC–LUR (10^−5^/m)	235	1.02 (0.92–1.14)	1.00 (0.90–1.11)
PM_10_–IDW (1 μg/m^3^)	241	1.17 (1.04–1.32)	1.18 (1.01–1.38)
SO_2_–IDW (1 μg/m^3^)	238	1.02 (0.97–1.07)	1.01 (0.95–1.06)

BC, black carbon.

a ORs per standardized increases (in parentheses), roughly corresponding to interquartile range.

b Adjusted for infant sex, First Nations status, parity, maternal age, maternal smoking during pregnancy, month–year of birth, income (quintile-census), maternal education (quartile-census).

